# Safety and feasibility analysis of transvaginal extraperitoneal space anatomical sacrospinous ligament suspension in the treatment of moderate to severe pelvic organ prolapse: a retrospective comparative cohort study

**DOI:** 10.3389/fmed.2026.1832026

**Published:** 2026-09-08

**Authors:** Zhenyue Qin, Dan Song, Hui Mao, Zhiyong Dong, Jiming Chen

**Affiliations:** 1 Shaoxing Maternity and Child Health Care Hospital, Shaoxing, China; 2 Maternity and Child Health Care Affiliated Hospital, Shaoxing University, Shaoxing, China; 3 Department of Gynecology, The People’s Hospital of Wenshan Zhuang and Miao Autonomous Prefecture, Wenshan, Yunnan, China; 4 The Second Affiliated Hospital of Chongqing Medical University, Chongqing, China; 5 The Third Affiliated Hospital of Nanjing Medical University (The Second People’s Hospital of Changzhou), Changzhou, China

**Keywords:** extraperitoneal approach, pelvic organ prolapse, sacrospinous ligament fixation, single-port laparoscopy, transvaginal

## Abstract

**Background:**

Sacrospinous ligament fixation has become one of the important surgical procedures for treating pelvic organ prolapse. The traditional vaginal sacrospinous ligament fixation determines the suspension site through digital rectal examination, which carries a higher risk of intraoperative nerve injury and hemorrhage. This article aims to introduce the transvaginal extraperitoneal space anatomical sacrospinous ligament fixation and compare it with the traditional vaginal sacrospinous ligament fixation. A total of 40 patients were included in this study, and all patients were divided into the TEASLF group (*n* = 20) and the TSSLF group (*n* = 20) according to the principle of voluntary choice.

**Materials and methods:**

This article proposes the transvaginal extraperitoneal space anatomical sacrospinous ligament fixation, combining the advantages of precise endoscopic treatment with sacrospinous ligament fixation, and retrospectively analyzes the clinical data of 40 patients to evaluate this procedure.

**Results:**

The treatment effects of the two groups were comparable. The intraoperative hemorrhage volume in this procedure was significantly reduced compared to the traditional group, but the operation time was slightly longer than that of the traditional group. There were no statistically significant differences between the two groups in postoperative hospital stay, postoperative ventilation time, catheter removal time, improvement in POPQ scores, and improvement in PFIQ-7 and PFDI-20 scores.

**Conclusion:**

Transvaginal extraperitoneal space anatomical sacrospinous ligament fixation is safe and effective for treating pelvic organ prolapse. This procedure provides better exposure of the surgical field, allows suturing under direct vision, helps avoid vascular and neuronal injury, and enables faster and more convenient hemostasis. This procedure also aids junior physicians in understanding the anatomy of the sacrospinous ligament, facilitating clinical teaching and technical promotion.

## 1 Introduction

Pelvic Organ Prolapse (POP) is a common disease with multifactorial etiology. The International Continence Society (ICS) and the International Urogynecological Association (IUGA) define it as “the descent of the anterior vaginal wall, posterior vaginal wall, uterus (cervix uteri), or vaginal apex (vaginal vault or cuff scar after hysterectomy) (1).” With increasing age and other risk factors, the probability of women developing POP also increases. Studies have shown that 4.1% of POP patients over 80 years old present with clinical symptoms (2, 3). Clinicians have also designed different surgical procedures to treat POP and improve its symptoms. In 1992, Delancey proposed the “three-level” theory of vaginal support structures (4), making surgical procedures for POP more precise. Since Sederl invented the sacrospinous ligament fixation (SSLF) in the 1950s, it has gradually evolved and improved to become one of the important surgical procedures for treating POP (5). Due to factors such as poor surgical field exposure and difficulty in precisely exposing the sacrospinous ligament during transvaginal sacrospinous ligament fixation (TSSLF), our team combined transvaginal single-port laparoscopy with TSSLF (6). The combined procedure not only utilizes the strong sacrospinous ligament for suspension but also allows direct visualization and dissection of the sacrospinous ligament under transvaginal single-port laparoscopy, thereby reducing injury and improving surgical precision. We demonstrate important anatomical structures through surgical images to illustrate how to dissect the sacrospinous ligament via the extraperitoneal space, and compare this procedure with TSSLF in a retrospective cohort study to evaluate its safety and feasibility.

## 2 Materials and methods

### 2.1 General data

**Figure 1:**
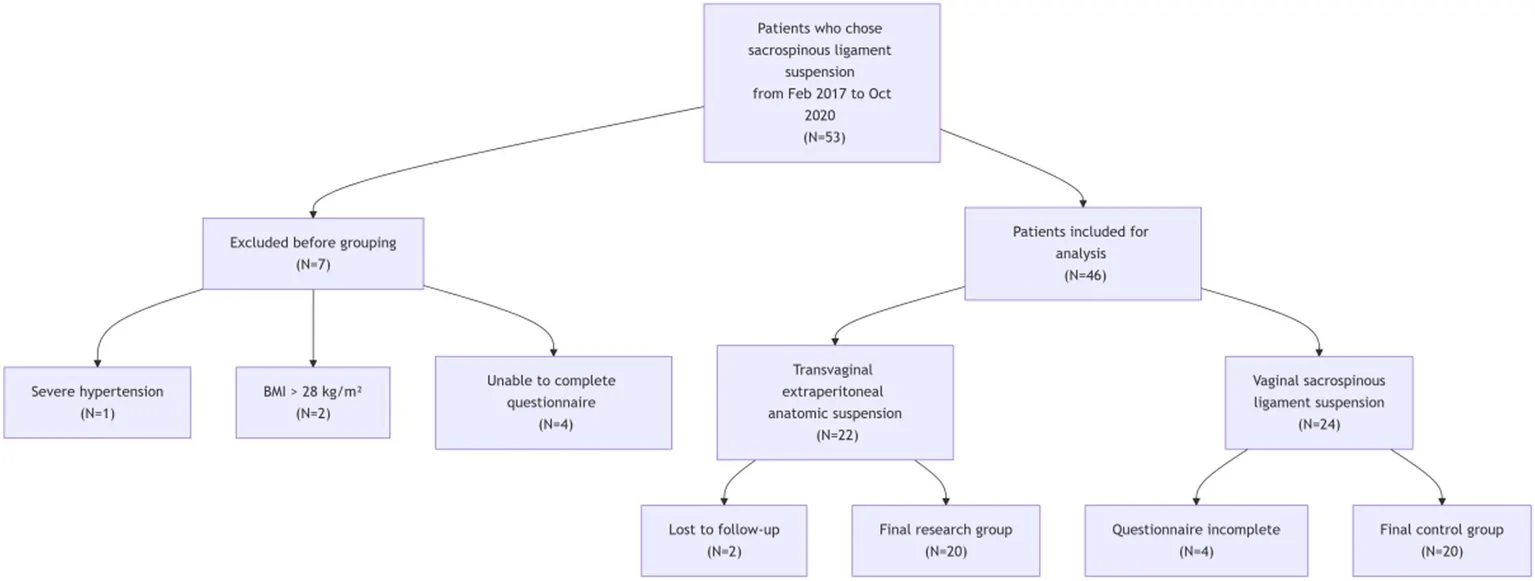
Patient enrollment and treatment flowchart for this study.

A retrospective cohort study was conducted to collect clinical data from 40 patients who underwent surgical treatment for POP in the Department of Gynecology, Changzhou Second People’s Hospital Affiliated to Nanjing Medical University from February 2017 to October 2020. All patients included in this study were diagnosed with stage II-IV POP based on POPQ scores after preoperative gynecological examination, and had complete preoperative Pelvic Floor Distress Inventory-Short Form 20 (PFDI-20) scores (7) and Pelvic Floor Impact Questionnaire-Short Form 7 (PFIQ-7) scores (7). According to the patients’ informed consent and voluntary choice of surgical method, they were divided into a study group (20 cases undergoing transvaginal extraperitoneal space anatomical sacrospinous ligament suspension, TEASLF) and a control group (20 cases undergoing TSSLF). This study was approved by the Medical Ethics Review Committee of Changzhou Second People’s Hospital Affiliated to Nanjing Medical University (No. [2017]-Y-15-01) (see Figure 1).

#### 2.1.1 Inclusion criteria

1. Preoperative diagnosis of stage II-IV POP according to POP-Q scoring (8).
2. Body Mass Index (BMI) < 28 kg/m^2^;
3. Complete medical records;
4. Ability to understand and complete the questionnaires;
5. Willingness and ability to attend regular postoperative follow-up visits;
6. Exclusion of patients with endometrial lesions;
7. Absence of severe internal medical or surgical comorbidities;
8. Surgery performed by the same team of medical personnel.

#### 2.1.2 Exclusion criteria

1. Patients with severe medical complications (referring to American Society of Anesthesiologists classification ≥ Grade IV, or uncontrolled severe hypertension, severe cardiac insufficiency, recent myocardial infarction, or other diseases that may affect the safety of surgical anesthesia) who cannot undergo surgery and anesthesia;
2. Patients with contraindications to surgery (e.g., acute reproductive tract infection, vaginal injury, vaginal stenosis, or other reproductive tract malformations);
3. Patients with pelvic or abdominal organ malignancies;
4. Patients lost to follow-up after treatment;
5. Patients unable to cooperate with questionnaire surveys.

## 3 Methods

### 3.1 Preoperative preparation

Preoperative laboratory tests and relevant auxiliary examinations (imaging studies and cervical liquid-based cytology) were completed to exclude contraindications. Vaginal disinfection was performed for 3 days prior to surgery to reduce the risk of postoperative infection. A liquid diet was initiated 2–3 days before surgery. Patients fasted for at least 12 h and abstained from water for at least 8 h preoperatively. Skin preparation was performed 1 day before surgery.

### 3.2 Surgical instruments and materials

Study Group: Stryker complete digital laparoscopic system, transvaginal single-port protective cover and dedicated port, one pair of conventional laparoscopic scissors, one needle holder, one ultrasonic scalpel, one suction device, one bipolar electrocoagulation forceps, two surgical dissecting forceps, one 30° conventional laparoscopic lens, one light source system and insufflation system, one set of conventional surgical instruments, two Ethibond Excel W6937 non-absorbable sutures, along with other absorbable sutures, silk threads, etc.

Control Group: Conventional transvaginal surgical instruments.

### 3.3 Anesthesia and positioning

Tracheal intubation general anesthesia was used. Before anesthesia, circulating nurses assisted patients into the lithotomy position (head low, feet high ≥30°, leg abduction <90°). The buttocks were positioned about one fist’s breadth beyond the edge of the operating table to provide space, and shoulder braces were placed to prevent slippage injury.

### 3.4 Surgical procedure

Control Group (TSSLF): The labia minora were sutured and fixed to the lateral aspects of the labia majora. The cervix was grasped with Allis forceps and pulled downward. Normal saline (10–20 mL) was injected into the vesicovaginal space to create a hydrodissection cushion. The vaginal wall was incised at the vaginal vault. A longitudinal incision was made in the anterior vaginal wall between the bladder transverse groove and the anterior vaginal fornix down to the hydrodissection plane. The protruding bladder was dissected. The defective bladder support tissues were plicated with purse-string and U-shaped sutures. Excess vaginal cuff was trimmed, and the vaginal incision was closed. A longitudinal incision was made in the posterior vaginal wall mucosa approximately 1 cm inside the hymenal caruncles. The posterior vaginal wall mucosa was bluntly dissected bilaterally to expose the rectovaginal septum and enter the pararectal space. After fully retracting the rectum, the ischial spine was identified. The sacrospinous ligament was exposed via dissection from the direction of the ischial spine. Two interrupted sutures using non-absorbable suture material were placed on the right sacrospinous ligament, about two fingerbreadths medial to the ischial spine. The other ends of the sutures were attached to the submucosa of the vaginal apex. After tying the knots, the vagina was elevated. Finally, the incision was routinely closed with absorbable sutures. After postoperative vaginal disinfection, an iodophor gauze was placed and removed the next day. (Note: In this procedure, hysterectomy was performed prior to the suspension; hysterectomy steps are omitted.)

**Figure 2:**
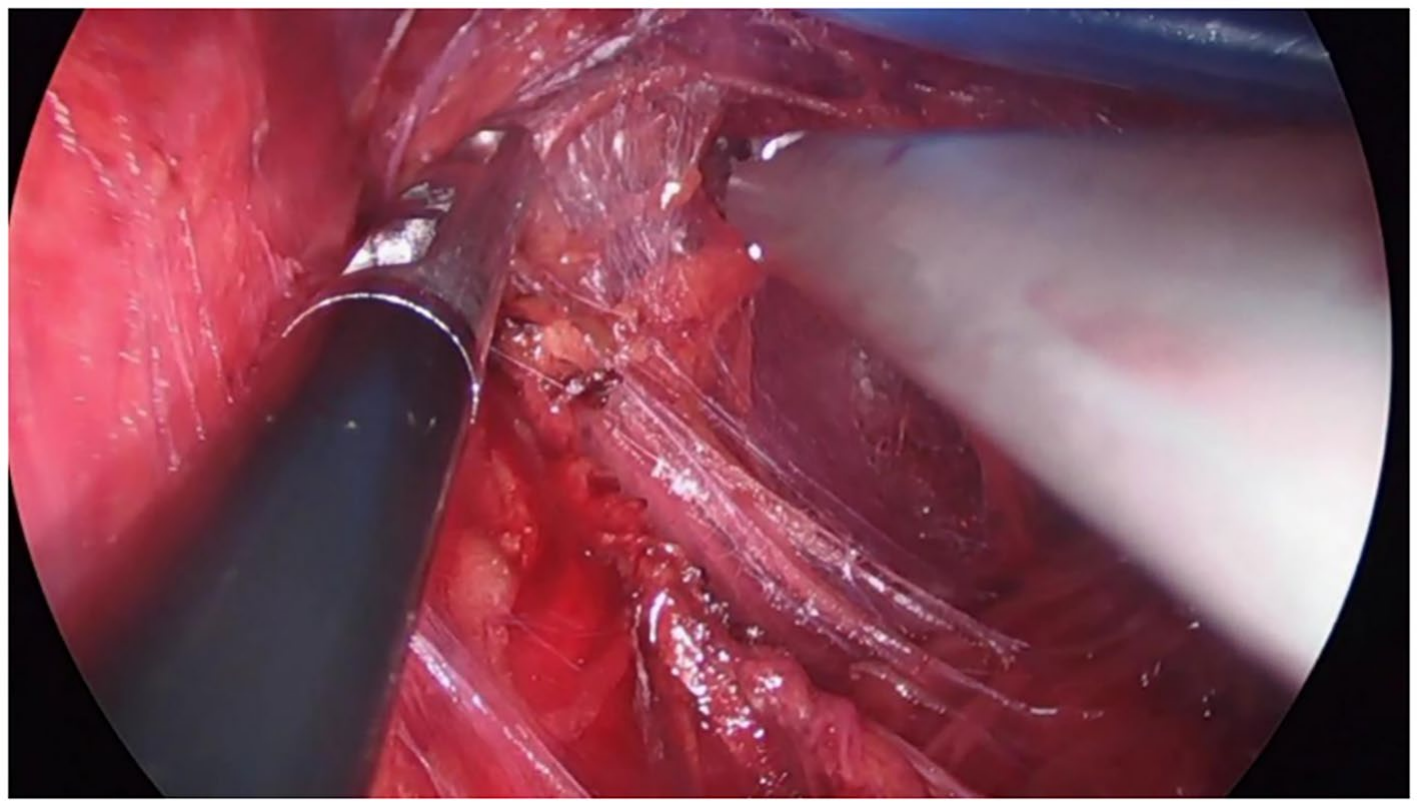
Dissecting the space.

**Figure 3:**
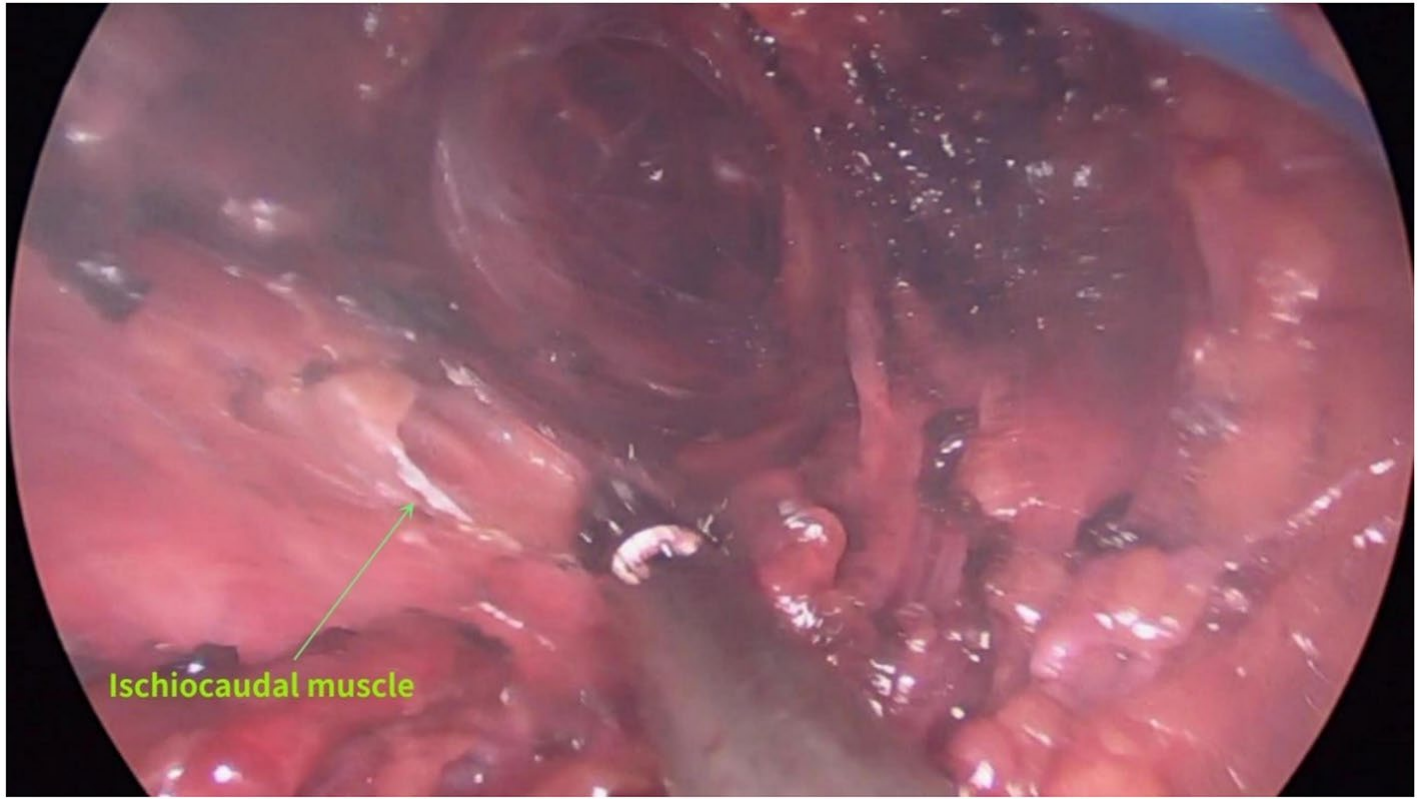
Freeing the fascia overlying the coccygeus muscle.

**Figure 4:**
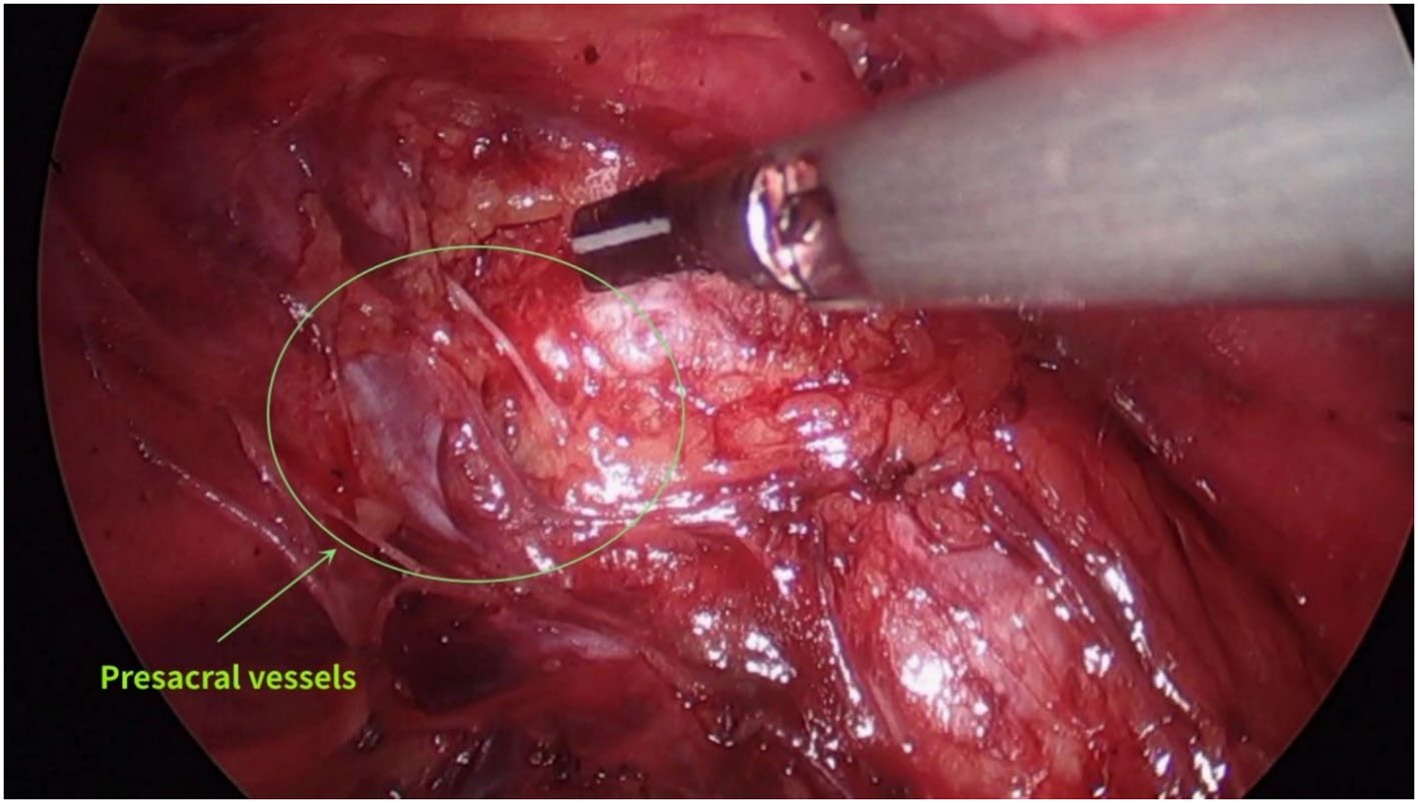
Dissecting the presacral vessels.

**Figure 5:**
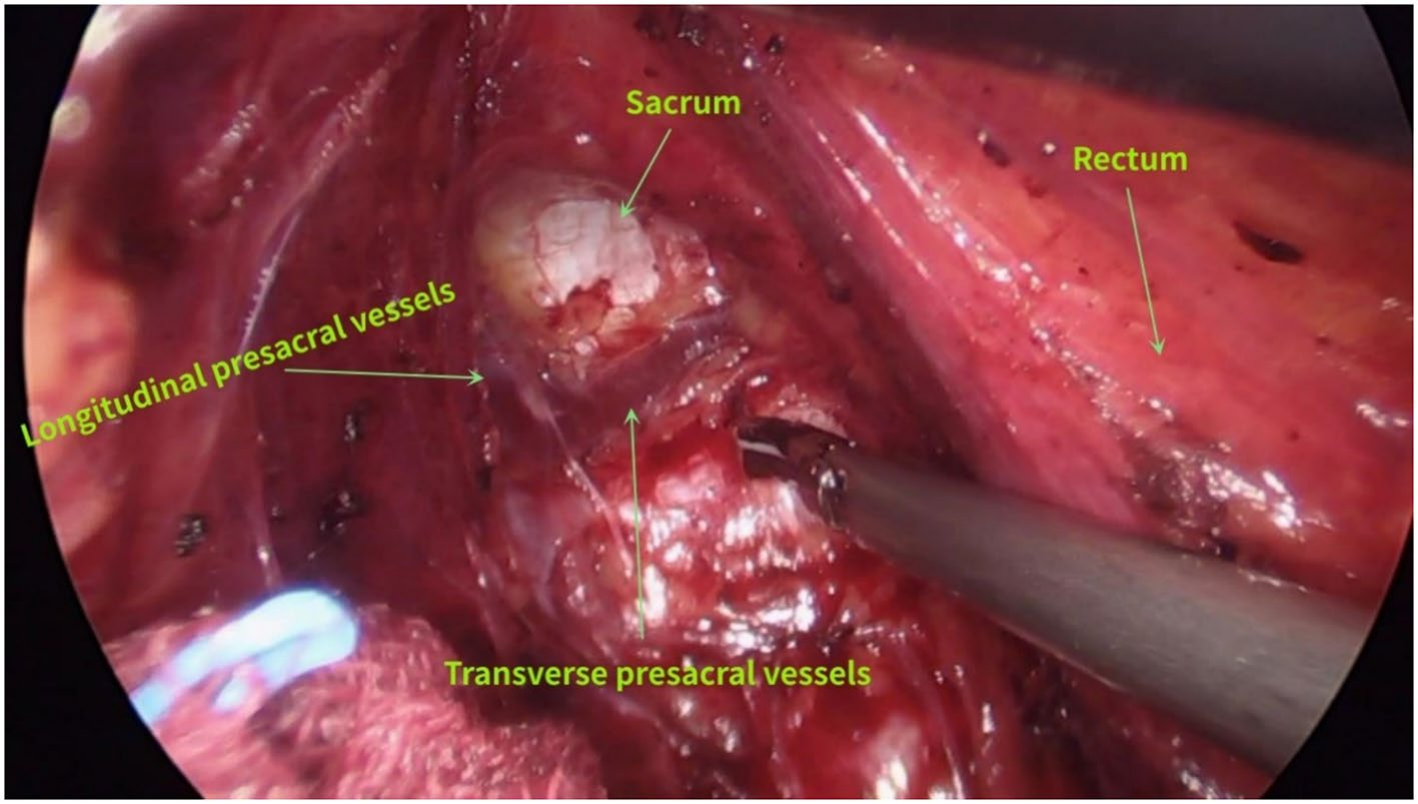
Presacral transverse and longitudinal vessels.

**Figure 6:**
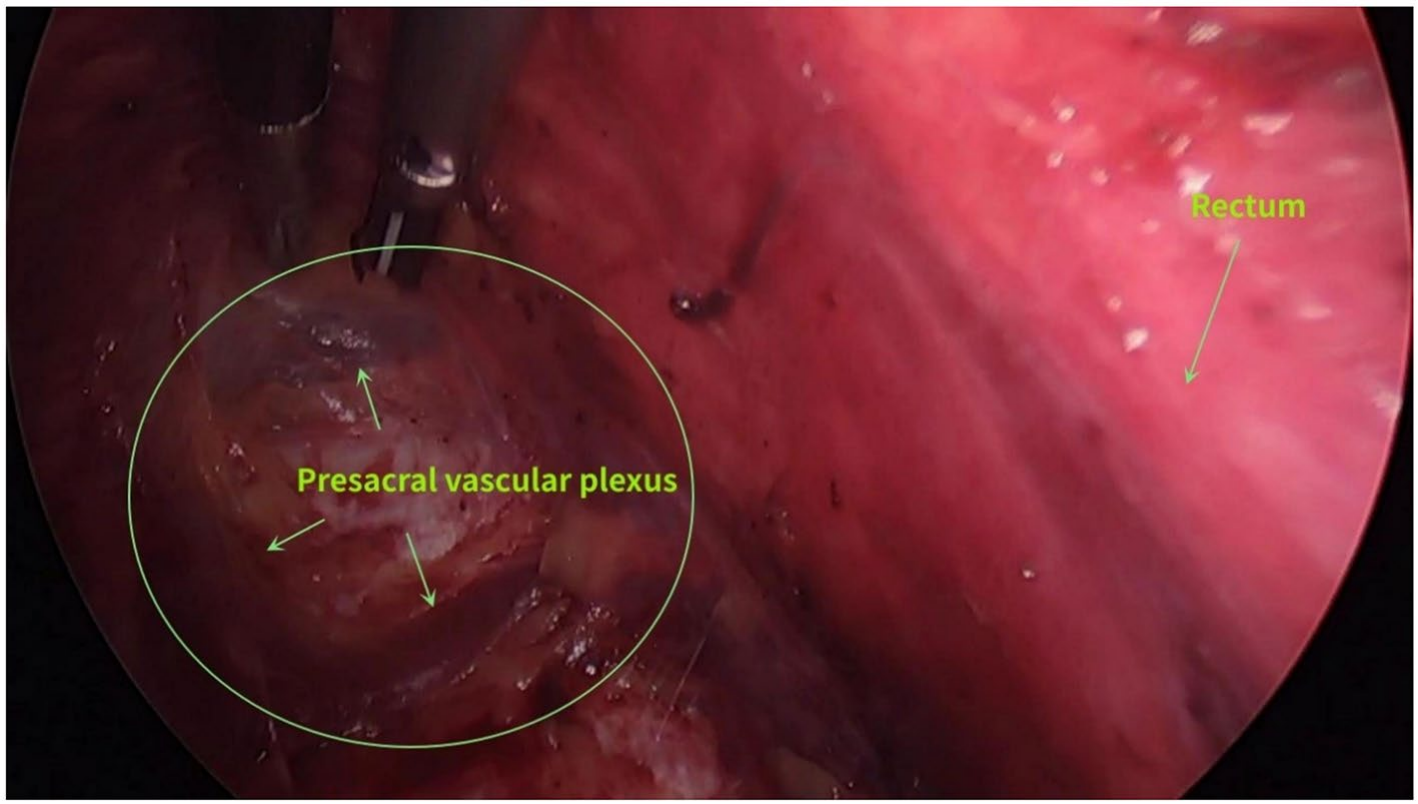
Presacral vascular plexus.

**Figure 7:**
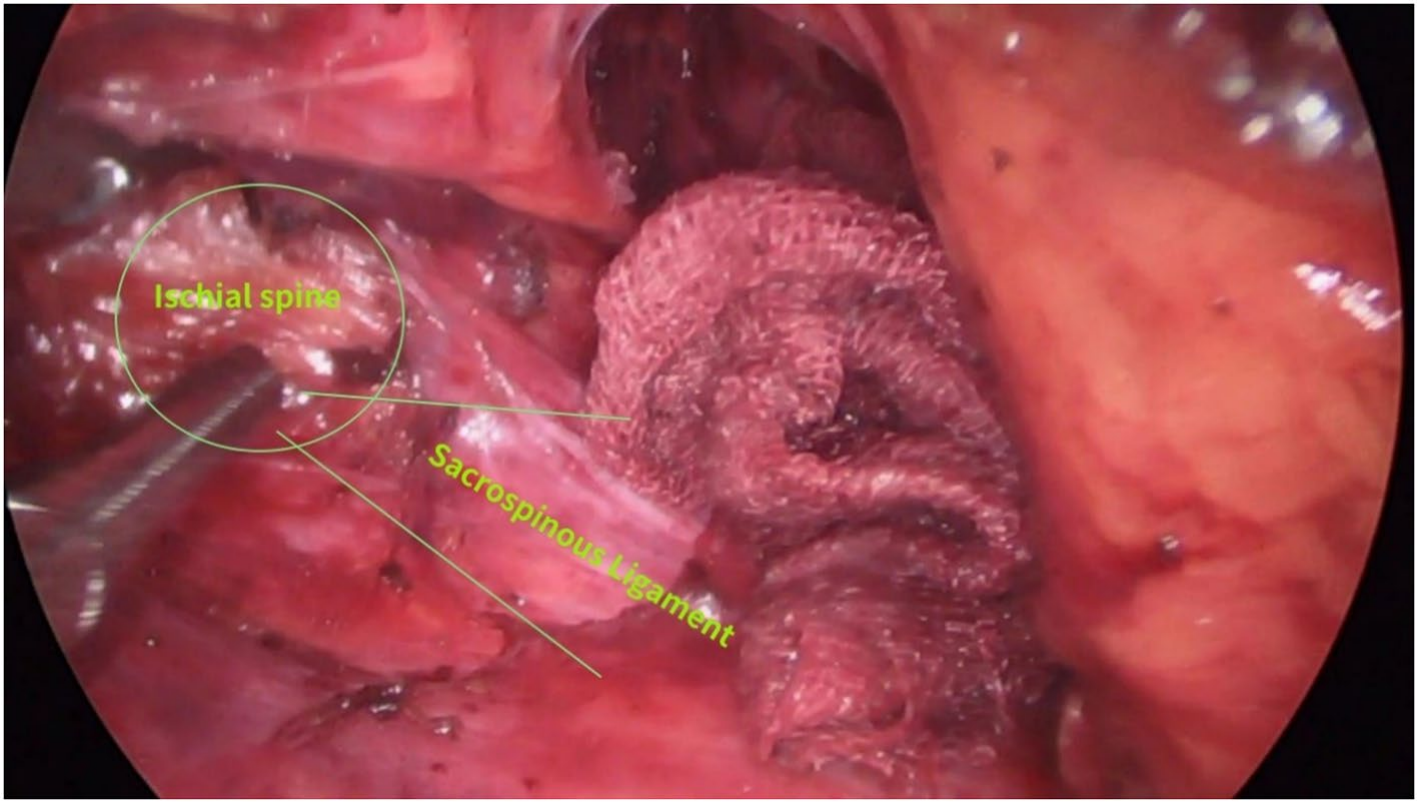
Dissecting the ischial spine and sacrospinous ligament.

**Figure 8:**
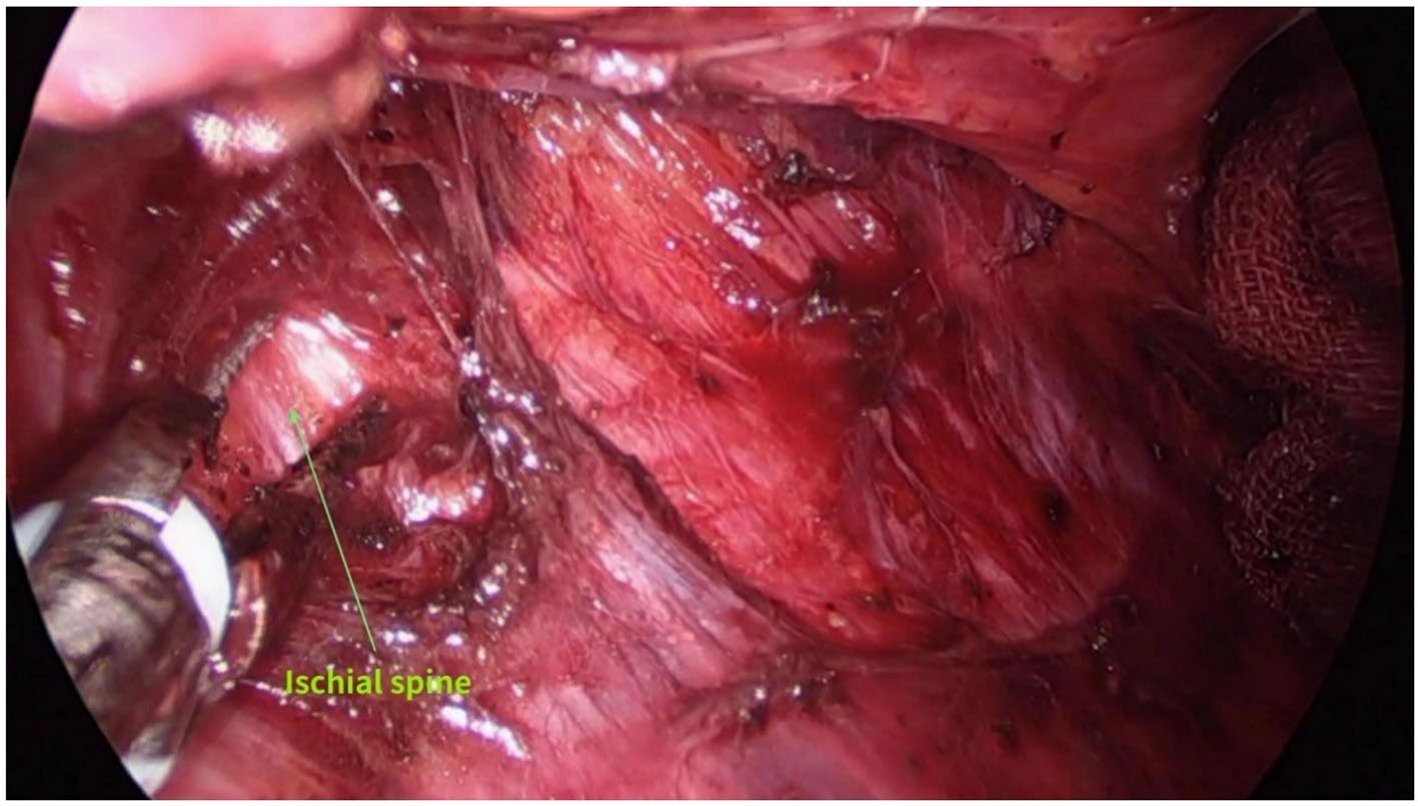
Ischial spine.

**Figure 9:**
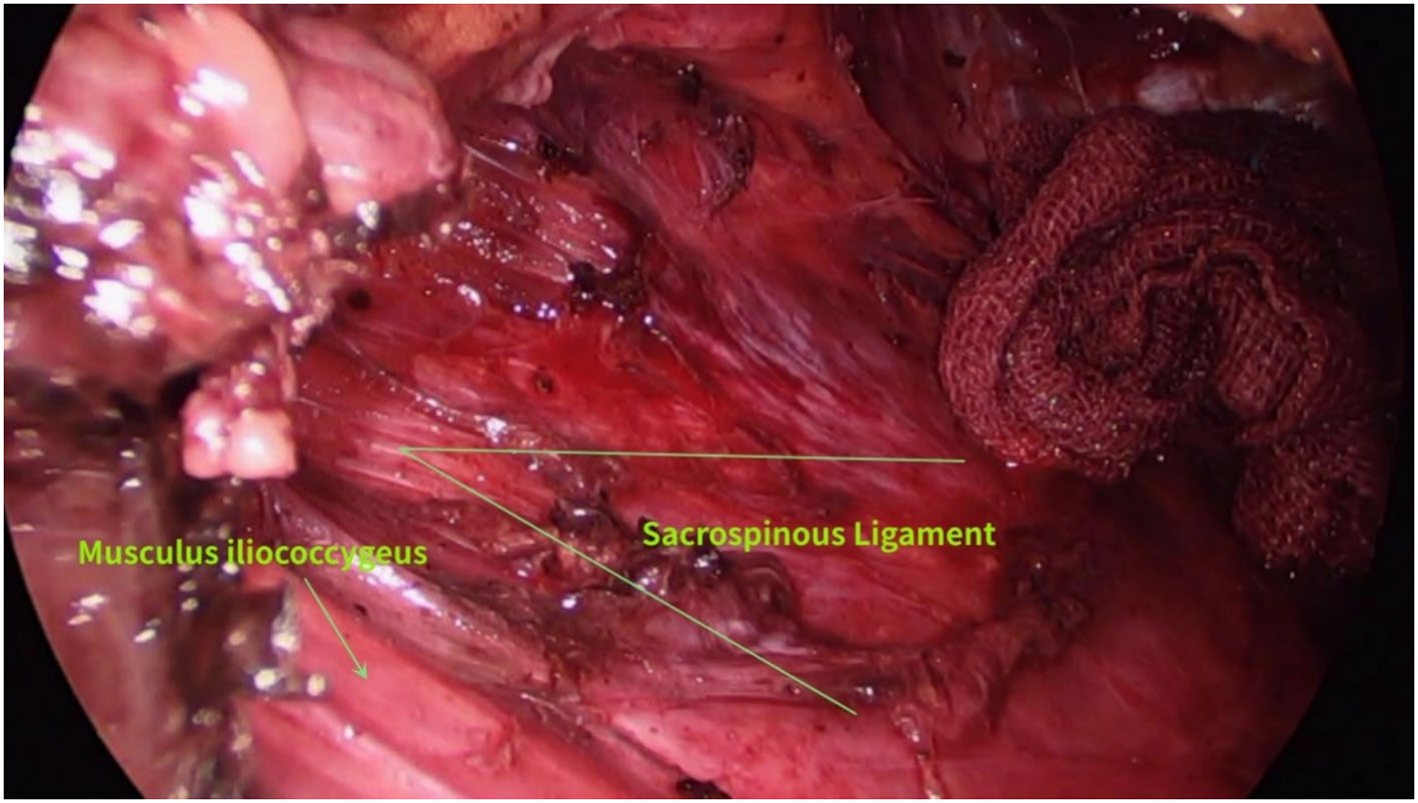
Iliococcygeus muscle and sacrospinous ligament.

**Figure 10:**
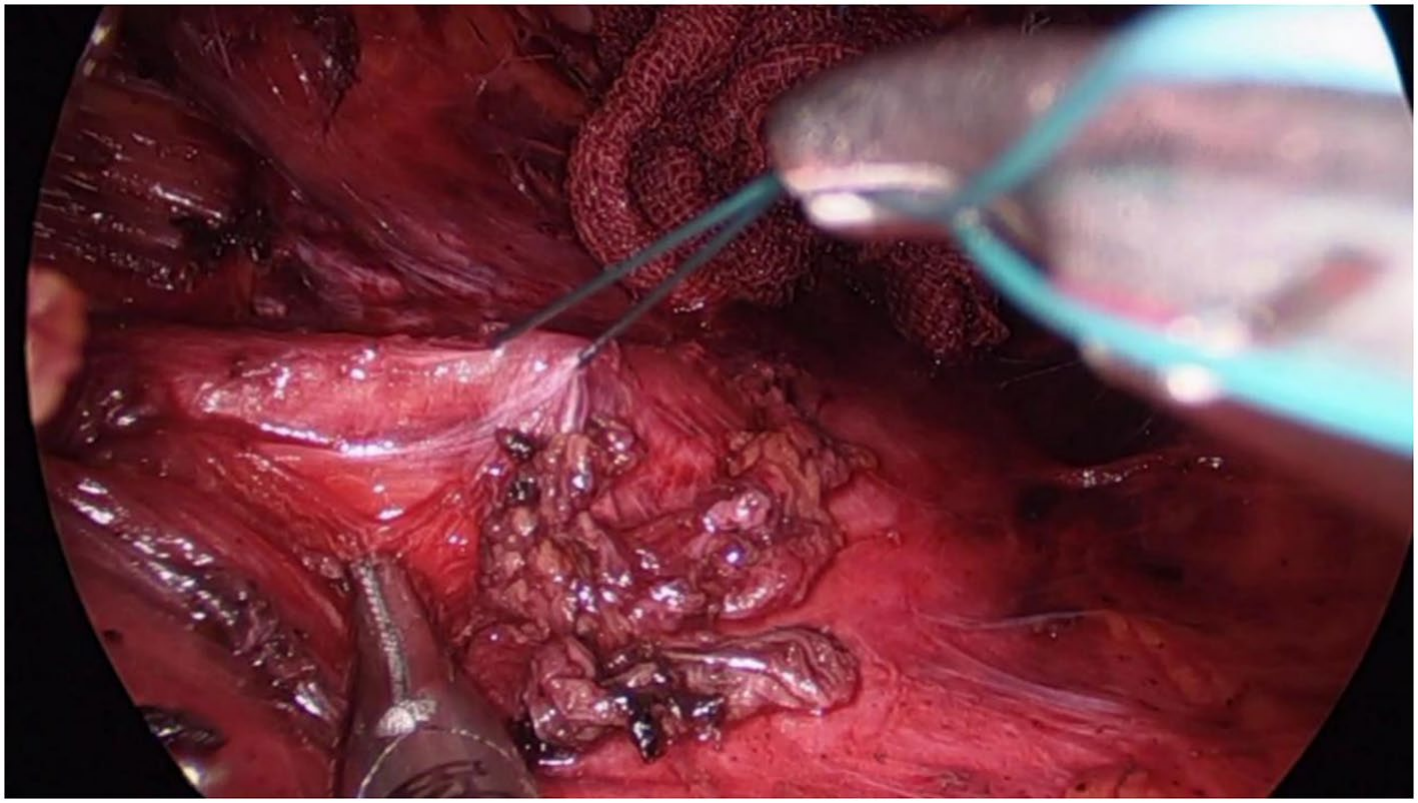
Testing the tension of the sacrospinous ligament.

Study Group (TEASLF): After exposing the surgical field, a longitudinal incision of about 2.0–3.0 cm was made in the mid-to-lower segment of the posterior vaginal wall. A small, confined potential space was initially created by finger dissection, while simultaneously pushing the rectum towards the patient’s left side as much as possible to avoid intraoperative complications. A purse-string suture was placed around the wound edges (to prevent gas leakage after placing the wound protector and tightening the purse-string). A single-port wound protector was placed within the purse-string to dilate the incision and create a sealed surgical workspace. The transvaginal single-port laparoscopic dedicated port was connected to the wound protector. The insufflation platform was connected, and CO₂ gas was insufflated to a pressure of 11 mmHg (1 mmHg = 0.133 kPa) to establish pneumoperitoneum. A 30° laparoscope was inserted through the port to visualize the field. Dissecting forceps were used to retract the bowel to avoid injury. An ultrasonic scalpel was used to meticulously dissect surrounding connective tissue to further enlarge the space (see Figure 2). The fascia overlying the coccygeus muscle was freed (see Figure 3). For beginners, anatomical structures can be dissected step-by-step until the sacrum and the locations of surrounding vessels and nerves are identified, allowing visualization of the presacral longitudinal and transverse vessels (see Figures 4, 5). Attention was paid to the distance between the presacral vascular plexus and the rectum (see Figure 6). Every effort was made to avoid injuring vessels and nerves to minimize patient trauma. With proficiency in this technique, extensive tissue exposure is not necessary to locate the sacrospinous ligament. Bleeding during space dissection was controlled with gauze packing. Further dissection exposed the ischial spine and the iliococcygeus muscle (see Figures 7, 8). Landmark anatomical structures of the pelvic floor, such as the sacrum, coccygeus muscle, ischial spine, inferior gluteal vessels, and sciatic nerve, were used to locate and expose the sacrospinous ligament for suture placement (see Figure 9). After confirming the location, the suture site could be marked using bipolar coagulation. Two Ethibond Excel W6937 non-absorbable sutures were used to place two stitches through the sacrospinous ligament. The suture depth was approximately half the ligament’s thickness, with about 1 cm between stitches. After needle placement, the tension was assessed by pulling on the sutures with dissecting forceps (see Figure 10). Knots were not tied at this stage. The port was removed. The other ends of the non-absorbable sutures were tied to the sutures at the vaginal apex in patients who underwent hysterectomy, or to sutures placed approximately 3 cm below the external cervical os (near the attachment of the uterosacral ligaments to the cervix) in patients who underwent vaginal wall repair without hysterectomy. After knot tying, the cervix was grasped with a tenaculum and lifted to check if it was fixed at the level of the sacrospinous ligament. The posterior vaginal wall incision was then sutured to conclude the procedure. After postoperative vaginal disinfection, an iodophor gauze was placed and removed the next day. (Note: In this procedure, the suspension was performed prior to hysterectomy; hysterectomy steps are omitted.)

### 3.5 Postoperative care

Patients were safely returned to the ward. They remained flat in bed without a pillow for 6–8 h to prevent headaches caused by decreased intracranial pressure. Continuous ECG and blood pressure monitoring was maintained for 24 h postoperatively, with close attention to vital signs, urinary catheter patency, urine output, and color. The urinary catheter was typically removed on postoperative day 5. Oral intake was strictly prohibited for the first 6 h postoperatively to prevent aspiration. Water intake was allowed after 6 h. Based on patient recovery, a liquid diet could be initiated 6–12 h postoperatively to promote gastrointestinal motility. Special attention was paid to the amount of vaginal bleeding. Patients were instructed to keep the perineal area clean and dry to prevent infection.

### 3.6 Observation indicators

#### 3.6.1 Preoperative indicators

Age (years), gravidity, parity, Body Mass Index (BMI), degree of uterine prolapse, POP-Q measurement points, PFDI-20 score, PFIQ-7 score.

Intraoperative Indicators: Operative time (minutes), intraoperative blood loss (ml).

Postoperative Indicators: Time to urinary catheter removal (days), time to first flatus (hours), postoperative hospital stay (days).

#### 3.6.2 Postoperative follow-up indicators

POP-Q measurement points, PFDI-20 score, and PFIQ-7 score were recorded postoperatively.

### 3.7 Statistical analysis

Statistical analysis was performed using R version 4.5.0 software. Normality was tested using the Shapiro–Wilk test. For normally distributed measurement data, results were expressed as mean ± standard deviation (x̄ ± s), and differences between two groups were compared using the independent sample t-test. For non-normally distributed measurement data, results were expressed as median (interquartile range), and the Mann–Whitney U test was used. Count data were expressed as number of cases (percentage), and intergroup comparisons were performed using the continuity-corrected chi-square test or Fisher’s exact test (when the expected frequency was <5). A two-sided *p*-value of <0.05 was considered statistically significant. This study did not pre-calculate sample size or perform power analysis, which is one of the limitations of the study.

## 4 Results

### 4.1 Basic characteristics of the study population

Initially, 53 patients who underwent SSLF for POP between February 2017 and October 2020 were considered. Thirteen were excluded for not meeting inclusion criteria (1 with severe hypertension, 4 unable to cooperate with questionnaires, 2 with BMI > 28 kg/m^2^, 6 lost to follow-up). Forty patients meeting the study criteria were enrolled: 20 voluntarily chose TSSLF and 20 voluntarily chose TEASLF. Comparisons of age, gravidity, parity, BMI, and degree of uterine prolapse between the two groups showed no statistically significant differences (*p* > 0.05), as shown in Table 1.

**Table 1:** Basic information of patients with different surgical procedures.

	Undergo SSLF (*N* = 40)		*p*
	TEASLF (*N* = 20)	TSSLF (*N* = 20)	
Age (years)	61.8 ± 10.7	60.35 ± 9.4	0.652
Gestation times			0.744
<3	7(35.0%)	8(40.0%)	
≥3	13(65.0%)	12(60.0%)	
Parturition times			1
<3	15(75.0%)	15(75.0%)	
≥3	5(25.0%)	5(25.0%)	
BMI	23.5 ± 1.7	24.2 ± 1.6	0.147
Stage of pelvic organ prolapse			0.520
II	11(55%)	13(65%)	
III	6(30%)	3(15%)	
IV	3(15%)	4(20%)	

Pregnancy, parity and Stage of pelvic organ prolapse are classified variables, expressed as *n* (%), the statistical method is chi-square test, age and BMI are continuous variables, expressed by mean ± standard deviation, and the statistical method is *t*-test.

### 4.2 Comparison of perioperative indicators between different surgical techniques

To investigate the impact of different techniques on postoperative outcomes, we obtained data on operative time, intraoperative blood loss, postoperative hospital stay, time to first flatus, and time to catheter removal. There was a statistically significant difference in operative time between the two groups (183.5 ± 45.10 vs. 122.6 ± 47.41, *p* < 0.001; operative time included hysterectomy time). Intraoperative blood loss was significantly lower in the TEASLF group compared to the TSSLF group (41.5 ± 26.21 vs. 59.8 ± 23.80, *p* = 0.026). There were no statistically significant differences in the other indicators (*p* > 0.05), as shown in Table 2.

**Table 2:** Comparison of perioperative conditions of different surgical procedures.

	Undergo SSLF(*N* = 40)		*t*	*p*
	TEASLF (*N* = 20)	TSSLF (*N* = 20)		
Operation time (min)	183.50 ± 45.10	122.60 ± 47.41	4.16	<0.001
Intraoperative bleeding volume (mL)	41.50 ± 26.21	59.80 ± 23.80	−2.31	0.026
Postoperative hospital stay (d)	6.40 ± 1.14	6.30 ± 1.65	0.22	0.825
Postoperative ventilation time (h)	23.3 ± 1.26	22.25 ± 2.44	1.71	0.096
Catheter removal time (d)	5.05 ± 1.39	4.5 ± 1.27	1.30	0.201

### 4.3 Comparison of POP-Q measurement points between the two groups

T-tests were performed on the measurement points (Aa, Ba, C, Ap, Bp) preoperatively and postoperatively (at 6, 12, and 24 months) for both groups. There were no statistically significant differences in these measurement points between the two groups preoperatively or postoperatively (*p* > 0.05), as shown in Table 3.

**Table 3:** Comparison of measurement indexes of each indicator point of POP-Q in patients with different surgical procedures before and after surgery (6, 12, and 24 months).

	Undergo SSLF (*N* = 40)		*t*	*p*
	TEASLF (*N* = 20)	TSSLF (*N* = 20)		
Preoperative				
Aa	1.03 ± 0.77	1.05 ± 0.72	−0.106	0.916
Ba	2.35 ± 0.97	2.25 ± 0.85	0.346	0.731
C	3.73 ± 0.47	3.75 ± 0.41	−0.178	0.860
Ap	1.48 ± 0.75	1.85 ± 0.73	−1.603	0.117
Bp	1.33 ± 0.98	1.20 ± 0.70	0.466	0.644
6 months after surgery				
Aa	−2.20 ± 0.25	−2.30 ± 0.25	1.258	0.216
Ba	−2.80 ± 0.25	−2.73 ± 0.34	−0.789	0.435
C	−7.20 ± 0.25	−7.23 ± 0.26	0.312	0.757
Ap	−2.18 ± 0.24	−2.30 ± 0.25	1.594	0.119
Bp	−2.88 ± 0.22	−2.83 ± 0.24	−0.677	0.503
12 months after surgery				
Aa	−2.00 ± 0.43	−1.95 ± 0.46	−0.357	0.723
Ba	−2.53 ± 0.38	−2.50 ± 0.43	−0.195	0.846
C	−6.95 ± 0.54	−6.8 ± 0.64	−0.806	0.425
Ap	−1.98 ± 0.41	−1.98 ± 0.44	0.000	1.000
Bp	−2.45 ± 0.39	−2.15 ± 1.16	−1.095	0.280
24 months after surgery				
Aa	−1.70 ± 0.55	−1.65 ± 0.56	−0.284	0.778
Ba	−2.30 ± 0.34	−2.33 ± 0.37	−0.222	0.826
C	−6.63 ± 0.79	−6.60 ± 0.64	−0.110	0.913
Ap	−1.70 ± 0.55	−1.68 ± 0.57	−0.142	0.888
Bp	−2.28 ± 0.34	−2.35 ± 0.37	0.668	0.508

### 4.4 Comparison of PFDI-20 and PFIQ-7 scores between the two groups

Comparisons of PFDI-20 and PFIQ-7 scores preoperatively and postoperatively (at 6, 12, and 24 months) revealed no statistically significant differences between the two groups at any time point (*p* > 0.05), as shown in Table 4.

**Table 4:** Comparison of PFDI-20 and PFIQ-7 in patients with different surgical procedures before and 6, 12, and 24 months after operation.

	Undergo SSLF(*N* = 40)		*t*	*p*
	TEASLF (*N* = 20)	TSSLF (*N* = 20)		
Preoperative				
PFDI-20	109.30 ± 4.81	110.10 ± 5.09	−0.511	0.612
PFIQ-7	103.80 ± 2.97	105.20 ± 2.80	−1.534	0.133
6 months after surgery				
PFDI-20	29.30 ± 4.78	30.60 ± 3.73	−0.959	0.344
PFIQ-7	30.20 ± 3.43	29.00 ± 1.65	1.410	0.167
12 months after surgery				
PFDI-20	29.70 ± 3.87	28.60 ± 2.91	1.016	0.316
PFIQ-7	28.50 ± 2.59	28.60 ± 3.90	−0.096	0.924
24 months after surgery				
PFDI-20	29.40 ± 3.19	30.30 ± 2.92	−0.931	0.358
PFIQ-7	30.60 ± 3.12	30.90 ± 3.28	−0.297	0.768

### 4.5 Perioperative complications

This study systematically reviewed and recorded the perioperative complications in both groups of patients, focusing on predefined events such as vascular injury, neuronal injury (including pudendal neuropathy, gluteal/gluteal muscle pain, sciatica), pelvic organ injury (Bladder, Rectum, Ureter), postoperative dysuria, new-onset urinary incontinence, incision infection, and Deep venous thrombosis. The results showed that no such complications were recorded intraoperatively or postoperatively in either the TEASLF group or the TSSLF group (Table 5). No cases required reoperation in either group.

**Table 5:** Comparison of perioperative complications between the two groups (cases).

Complications	TEASLF (*N* = 20)	TSSLF (*N* = 20)
Vascular injury	0	0
Neuronal injury (gluteal region/sciatic nerve/pudendal neuropathy)	0	0
Bladder injury	0	0
Rectal injury	0	0
Ureteral injuries	0	0
Postoperative dysuria/urinary retention	0	0
Postoperative new-onset urinary incontinence	0	0
Incision infection	0	0
Deep vein thrombosis	0	0

Since none of the above complications occurred in either group, no intergroup statistical tests were performed.

## 5 Discussion

With increasing female age, the incidence of POP is also rising annually. POP has multiple risk factors, including age, hysterectomy, obesity (BMI > 30 kg/m^2^), smoking, chronic Valsalva maneuvers (coughing, straining, heavy lifting), multiparity and history of vaginal delivery, and genetic defects in pelvic floor support (9). For asymptomatic patients, lifestyle modifications such as avoiding heavy lifting and high-intensity exercise, treating constipation, and quitting smoking can delay disease progression. For patients with mild symptoms who decline surgery, conservative treatments like pelvic floor muscle exercises (Kegel exercises) (10), estrogen therapy (11), and pessaries are options. For patients with significant clinical symptoms, surgical intervention can better alleviate symptoms, with approximately 13% of women undergoing prolapse surgery in their lifetime (12).

Numerous surgical techniques exist for treating POP. TSSLF as one of the classic procedures (13), offers advantages including (1) firm fixation, durable efficacy, and low recurrence rates; (2) minimal physical impact on the patient and rapid postoperative recovery; and (3) preservation of vaginal depth and satisfactory restoration of sexual function. Some reports suggest an effectiveness rate of up to 90% for treating POP (14). However, this procedure carries several potential complications, such as fever, bleeding, injury to the femoral, peroneal, and sciatic nerves, gluteal pain, bladder pain, injury to other pelvic organs, vaginal adhesions, or rectovaginal fistula (13). Analyzing the surgical process, we believe the cause of complications may be attributed to poor exposure of the surgical field. With the traditional transvaginal approach, surgeons rely on experience and manual palpation to locate the ischial spine and subsequently the sacrospinous ligament. This process may lead to inaccurate or erroneous anchor point placement, potentially increasing surgical recurrence rates or even causing failure (15).

In recent years, with advancements in endoscopic technology and the growing pursuit of minimally invasive techniques, novel minimally invasive endoscopic approaches have flourished and gained patient preference. The concept of Natural Orifice Transluminal Endoscopic Surgery (NOTES) was first proposed by foreign scholar Peter Wilk in 1994. In 1998, Moran provided a detailed description of specific operative pathways based on this concept. Marescaux et al. performed the first clinical transvaginal endoscopic cholecystectomy, marking the official entry of NOTES into clinical practice (16). In the field of gynecology, this technique has also been widely applied (17–19). In 2019, Liu et al. (20) reported a clinical study of 26 cases of transvaginal endoscopic sacrocolpopexy, successfully completing 23 procedures with significant improvement in patients’ quality of life and no complications such as pain, hematoma, infection, or new-onset urinary incontinence. Building upon the maturity of both SSLF and transvaginal single-port laparoscopic surgery, we integrated transvaginal NOTES with SSLF, combining their advantages. This approach utilizes the robust sacrospinous ligament while incorporating the benefits of laparoscopic visualization and precision. Other scholars have also confirmed the feasibility and effectiveness of transvaginal single-port laparoscopic sacrospinous ligament fixation for POP (16, 21, 22).

Intraoperative identification of the sacrospinous ligament and selection of the appropriate puncture site are crucial. Deviation in puncture site selection may lead to postoperative complications like pain (23). Some scholars suggest that visualizing the puncture site can reduce surgical complications to some extent (24). This highlights the importance of thorough knowledge of female pelvic floor anatomy for the surgeon. One study noted that nerves to the coccygeus and levator ani course over the midportion of the C-SSL where SSLF sutures are placed. The pudendal nerve and inferior gluteal artery are in proximity to the superior border of the C-SSL at its midportion, while the internal pudendal artery passes behind the ischial spine, lateral to the recommended site for suture placement (25). Another scholar also emphasized that knowledge of SSL anatomy is crucial, and during surgical training, it is clearly indicated to stay far away (almost 2 cm) from the ischial spine to avoid nerve and vascular injuries (26). Thus, the success of the surgery hinges critically on the choice of the suspension point. For improving surgical proficiency, models have been developed to facilitate learning SSLF (27, 28). However, individual patient anatomical variations exist, while models are standardized. Laparoscopic visualization allows for clearer observation of diverse pelvic floor structures, making it more conducive to learning for junior physicians (29), which is also one of the advantages of the study group.

All TEASLF and TSSLF surgeries in this study were performed by the same surgical team. The lead surgeon had over 10 years of experience in pelvic floor surgery, had completed more than 200 SSLF procedures, and was at a stable plateau of technical proficiency. The first assistant involved in the surgeries was a senior attending physician who had completed standardized laparoscopic technology training during the data collection period. It should be noted that this study did not systematically analyze the learning curve of TEASLF; therefore, the conclusion that “TEASLF facilitates the teaching of junior physicians” is primarily based on the subjective teaching experience of the surgeons and lacks quantitative indicators for support (e.g., trends in surgical time across different learning stages, changes in complication rates, etc.). It is recommended that future studies incorporate learning curve analysis to objectively evaluate the teaching value of this technique.

The data in this study were derived from 40 patients. As shown in Table 1, there were no significant differences in baseline characteristics including age, BMI, gravidity, parity, and degree of uterine prolapse between the two groups. In Table 2, the operative time was significantly longer in the study group (183.50 ± 45.10 min) than in the control group (122.60 ± 47.41 min) (*p* < 0.05), while intraoperative blood loss was significantly lower in the study group (41.50 ± 26.21 mL) than in the control group (59.80 ± 23.80 mL), with statistical significance (*p* < 0.05). This is consistent with data from two other scholars (16, 22). The longer operative time in the vNOTES group compared to the TSSLF group is attributed to the inclusion of hysterectomy time as well as the time required for establishing and closing the laparoscopic access. In the vNOTES group, exposure and suturing of the sacrospinous ligament is performed first, followed by hysterectomy; the time for exposing and suturing the ligament alone takes only about 50 min. Comparisons of postoperative catheter removal time, time to first flatus, and postoperative hospital stay showed no statistically significant differences between the two groups (*p* > 0.05). However, we observed that the postoperative hospital stay in our study group (6.40 ± 1.14 days) was longer than reported in two other studies (4 ± 2 days (16); 4.81 ± 1.25 days (22)). The postoperative stay in our control group (6.30 ± 1.65 days) was longer than one study (3 ± 1 day (16)) but similar to another (6.29 ± 2.38 days (22)). This difference may be related to the following factors: (1) The center in this study is a regional tertiary hospital, which accepts a large number of transferred patients from surrounding areas. Some patients need to be observed until no complications are confirmed before returning to their local area, objectively prolonging the length of hospital stay; (2) The urinary catheter removal time in this study was set at postoperative day 5 [different from the “catheter removal on postoperative day 1–2” protocol in other studies (16)], a relatively conservative strategy that may stem from the center’s cautious approach to postoperative urinary retention, but also directly extends the hospitalization time; (3) There are differences in postoperative management processes among domestic medical institutions. Some centers may not count the day of surgery when calculating hospital stay in days, whereas this study included the day of surgery in the total length of stay. These factors collectively led to a longer postoperative hospital stay in both groups of this study compared to some literature reports, reflecting the heterogeneity in real-world clinical practice rather than differences in the safety of the surgical technique itself.

From Table 3, we can observe no statistically significant differences in preoperative POP-Q measurement points between the two groups. Postoperative POP-Q measurements showed significant improvement compared to preoperative values in both groups, achieving anatomical correction. Comparisons of POP-Q measurement points at 6, 12, and 24 months postoperatively showed no statistically significant differences (*p* > 0.05), consistent with observations by other scholars (16). In Table 4, comparisons of preoperative PFDI-20 and PFIQ-7 scores between the study and control groups showed no statistically significant differences (*p* > 0.05). Postoperative PFDI-20 and PFIQ-7 scores significantly decreased compared to preoperative scores in both groups, demonstrating significant surgical efficacy. Comparisons at 6, 12, and 24 months postoperatively showed no statistically significant differences (*p* > 0.05). This conclusion aligns with one scholar’s findings (22), although another scholar reported that the pelvic function of the SvNOTE group was significantly better than that of the SSLF group (16). No recurrences were observed in either group during follow-up up to 24 months postoperatively.

(Recurrence is defined as: any indicator point of the POP-Q score decreasing by ≥2 grades compared to the measurement at 6 months postoperatively, or the occurrence of vaginal apex/anterior wall/posterior wall prolapse requiring re-surgical intervention, or the patient complaining of “something falling out” due to prolapse symptoms). Only one patient in the control group was found to have inflammatory granulation tissue growth (1*0.8*0.5 cm) at the vaginal cuff during a 6-month postoperative gynecological examination, which improved after symptomatic treatment. This was considered possibly related to individual reaction to suture material rather than the surgery itself.

The intraoperative blood loss in the TEASLF group was significantly lower than that in the TSSLF group. The reasons are analyzed as follows: (1) Due to the complex anatomical structure around the sacrospinous ligament, involving many nerves, internal pudendal artery, sacral plexus vessels, and ureter, the transvaginal single-port laparoscopic approach can overcome the drawbacks of the traditional transvaginal approach, expand the surgical field of view, and make the surgical process visible, which may help avoid vascular injury. (2) When a small amount of bleeding is detected during surgery, the surgeon can quickly achieve hemostatic under direct vision, thereby reducing intraoperative blood loss. However, it must be emphasized that no definite nerve injury events occurred in the TEASLF group in this study. The conclusion that “TEASLF can better protect the nerve” is still a theoretical inference at the technical level and lacks direct support from neurotrophic or neurophysiological monitoring evidence. Clearly exposing nerve structures during surgery does not equate to complete protection of postoperative nerve function, as mechanisms such as traction injury, thermal injury, and local hematoma compression may still affect nerve function. Future studies should introduce neurophysiological monitoring (e.g., pudendal nerve motor evoked potentials) or systematic sensory/motor nerve function assessments to obtain more direct evidence. Meanwhile, the operation time in the TEASLF group was significantly longer than that in the TSSLF group. The reasons are analyzed as follows: (1) The TEASLF group involved two additional steps compared to the TSSLF group during the procedure, mainly requiring the establishment of a laparoscopic approach at the start of surgery and suturing the vaginal wall incision at the end. (2) Since the transvaginal single-port laparoscopic approach is still in the exploratory stage, surgeons need to perform the procedure patiently to avoid unnecessary injury, and combined with laparoscopy, they perform anatomical surgery under visualization, which may prolong the operation time. (3) As the transvaginal single-port laparoscopic approach can expand the surgical field of view, junior surgeons can become familiar with the pelvic floor anatomical structure under direct vision and fully understand the anatomical structure of this area, which may initially increase the operation time.

Of course, our study also has certain limitations that should be fully considered when interpreting the results.

First, this study adopted a retrospective non-randomized controlled design, where patients voluntarily chose the surgical method based on personal preference, which may introduce selection bias. Although the baseline characteristics (age, BMI, parity, POP-Q stage) of the two groups were balanced and comparable, it is undeniable that patients who chose TEASLF may have a higher acceptance of new technologies and more positive expectations of treatment outcomes, or surgeons may have subconsciously selected traditional procedures for patients with more mature surgical techniques. These unmeasured confounding factors may affect the postoperative functional evaluation results. Additionally, the retrospective design determines that this study cannot conduct a prospective, standardized systematic assessment of perioperative complications, and some minor complications (such as transient gluteal discomfort) may be underestimated due to incomplete documentation in medical records.

Second, the sample size of this study is small (only 20 cases per group), and no pre-study sample size estimation or power analysis was performed. Given that the differences in primary efficacy indicators (POP-Q scores, PFDI-20/PFIQ-7 scores) between the two groups were not statistically significant, without power analysis, we cannot rule out the possibility of a Type II error (false negative), meaning that clinically meaningful differences between the two groups may exist but were not detected due to insufficient sample size. Future prospective studies with larger sample sizes are needed to confirm the non-inferiority or superiority of the two surgical procedures.

Third, the maximum follow-up duration in this study was 24 months. Although previous studies have shown that recurrence after SSLF mostly occurs within 2–3 years postoperatively, long-term follow-up data of more than 5 years are more critical for comprehensively evaluating surgical outcomes and long-term complications (such as chronic avulsion at the sacrospinous ligament suture site, delayed mesh/suture-related complications, etc.). Our center is continuing to follow up the above patients and plans to report separately after accumulating 5-year follow-up data with an expanded case series.

Fourth, this study did not conduct a specific assessment of postoperative sexual function, bladder function, and intestinal function. Although PFDI-20 and PFIQ-7 are internationally recognized screening tools for pelvic floor dysfunction, their coverage of sexual dysfunction (such as dyspareunia, decreased libido) and pain-related symptoms is incomplete. Future studies should introduce sexual function scales (such as PISQ-12) and visual analog scale (VAS) for pain to conduct multidimensional assessments.

Fifth, all surgeries were performed by the same group of physicians with extensive experience in pelvic floor surgery, whose technical proficiency may be higher than that of general gynecologists. Therefore, the generalizability of these study results is limited, especially during the learning curve phase of this technique, where complication rates and operative times may differ from the data reported in this study.

In summary, operators of laparoendoscopic single-site surgery must possess high professional competence; they must be proficient in laparoendoscopic single-site surgery techniques and have an in-depth understanding of pelvic floor anatomy. Future prospective, randomized controlled, multi-center studies will help further validate the safety and effectiveness of this technique and clarify its optimal indications.

## 6 Conclusion

For female patients with moderate to severe POP, both TEASLF and TSSLF demonstrate significant therapeutic effects, with comparable outcomes within the follow-up window of this study. However, TEASLF is associated with less intraoperative bleeding volume. This surgical approach improves the visual exposure of the operative field, thereby facilitating more precise anatomical identification and manipulation. The operative time for TEASLF is significantly longer than that for TSSLF. This difference may currently be attributed to the fact that the technique is still in an exploratory application stage, requiring sufficient time for meticulous anatomical dissection by the surgeon, and may also be related to the additional steps of establishing a laparoscopic approach and wound closure during the procedure. With the improvement of clinicians’ surgical skills and a thorough understanding of pelvic floor anatomy, the issue of prolonged operative time may be significantly alleviated in the future.

However, the above conclusions should be interpreted within the limitations of this study—its retrospective design, small sample size, lack of power analysis, and a two-year follow-up period all constrain the generalizability and certainty of the findings. The theoretical advantages of TEASLF in terms of neuroprotection require more direct clinical evidence for support. Future prospective, randomized controlled, large-sample, long-term follow-up studies are needed to further validate the clinical value of TEASLF compared with TSSLF.

## Data Availability

The data analyzed in this study is subject to the following licenses/restrictions: In-hospital internal data. Requests to access these datasets should be directed to ZQ, qinzy0622@163.com.
